# Effects of Hypoxia on Erythrocyte Membrane Properties—Implications for Intravascular Hemolysis and Purinergic Control of Blood Flow

**DOI:** 10.3389/fphys.2017.01110

**Published:** 2017-12-22

**Authors:** Ryszard Grygorczyk, Sergei N. Orlov

**Affiliations:** ^1^Medicine, Université de Montréal, Montreal, QC, Canada; ^2^Biology, M. V. Lomonosov Moscow State University, Moscow, Russia

**Keywords:** red blood cell, red cell ATP release, intravascular hemolysis, purinergic signaling, red cell membrane fragility, hypoxia-induced ATP release

## Abstract

Intravascular hemolysis occurs in hereditary, acquired, and iatrogenic hemolytic conditions but it could be also a normal physiological process contributing to intercellular signaling. New evidence suggests that intravascular hemolysis and the associated release of adenosine triphosphate (ATP) may be an important mechanism for *in vivo* local purinergic signaling and blood flow regulation during exercise and hypoxia. However, the mechanisms that modulate hypoxia-induced RBC membrane fragility remain unclear. Here, we provide an overview of the role of RBC ATP release in the regulation of vascular tone and prevailing assumptions on the putative release mechanisms. We show importance of intravascular hemolysis as a source of ATP for local purinergic regulation of blood flow and discuss processes that regulate membrane propensity to rupture under stress and hypoxia.

## Introduction

During the last three decades it has become increasingly clear that in addition to passive uptake and release of oxygen and metabolically-derived gases, the red blood cells (RBC) also exhibit diverse oxygen-sensitive responses that autonomously regulate their own properties and functions. For example, changes in partial oxygen tension (PO_2_) trigger a shift in glucose consumption from the pentose phosphate pathway (PPP) in oxygenated cells to glycolysis in deoxygenated cells (Messana et al., 1996). This shift is adaptive, since hemoglobin undergoes constant oxidation to methemoglobin in oxygenated cells, and its reduction back to hemoglobin would be facilitated by the enhanced production of NADPH in the PPP. Deoxygenation affects active Ca^2+^ transport and cytoplasmic Ca^2+^ buffering in human RBC (Tiffert et al., 1993). It was also shown that PO_2_ has an impact on the activity of RBC monovalent ion transporters (Bogdanova et al., 2009). In RBC from several fish species, low PO_2_ is required for β-adrenoceptor-mediated stimulation of Na^+^/H^+^ exchanger and elevation of hemoglobin affinity for O_2_ via cytoplasm alkalization (Nikinmaa, 2002). In human RBC with mutated hemoglobin (HbS) K^+^, Cl^−^ co-transport has an abnormal PO_2_-dependence that probably contributes to the pathogenesis of sickle cell anemia (Brugnara et al., 1996). Apart from the above-listed enzymatic and ion transport pathways, mammalian RBC also show hypoxia-induced responses involved in regulation of blood flow. These include two different, likely complementary mechanisms: rapid reduction of blood viscosity via increased RBC deformability and, delayed but sustained increase of vessel's diameter via release of adenosine triphosphate (ATP) and purinergic receptor stimulated production of NO and other vasorelaxants in vascular endothelial cells.

### Effects of hypoxia on blood viscosity and RBC deformability

Blood viscosity is determined by RBC flow properties that include adhesion, aggregation and deformability, i.e., ability to change shape under a given stress without hemolysing. Erythrocyte deformability affects blood flow in large blood vessels, due to the increased frictional resistance between fluid layers under laminar flow conditions. It also affects the microcirculatory blood flow significantly where erythrocytes are forced to pass through blood vessels with diameters smaller than their size. Numerous pathologies are associated with a decrease of RBC deformability. For our review it is important to note that increased blood viscosity in sickle cell anemia is caused by decreased RBC deformability due to gel formation of deoxygenated mutated hemoglobin HbS and its interaction with the cell membrane proteins determining membrane elasticity (for review see Yedgar et al., 2002; Diez-Silva et al., 2010; Viallat and Abkarian, 2014).

Modulation of blood viscosity by PO_2_ may involve different processes depending on the duration and extent of oxygenation/deoxygenation. On the long time scale, these may include gradual changes of RBC membrane surface charges, mainly due to reduction in sialic acid content, and deformability that correlate with age (Huang et al., 2011) and markers of oxidative stress (Mehdi et al., 2012). On the other hand, the impact of prolonged hypoxia on RBC membrane properties remains poorly defined which contrasts with well documented, e.g., altered protein sialation in other cells types such as tumor cells. However, brief dips to a lower range of PO_2_ as they occur in microcirculation, were recently found to have acute and significant impact on blood viscosity. Wei et al. (2016) reported that microinjection of O_2_ scavengers resulted in vasoactive mediator-independent capillary hyperemia in mice cerebral microcirculation. In additional experiments, using microfluidic channels of small (5 μm) or large (20 μm) size they assessed effect of oxygenation on erythrocyte flow velocity and shear-induced deformability, respectively. These experiments revealed that O_2_ depletion increased the velocity of erythrocyte flowing through the microfluidic channel due to increased RBC membrane deformability. Viewed collectively, these data demonstrate that in addition to the increment of vessel diameter (see below), elevation of blood flow in microcirculatory beds under hypoxic conditions might be achieved via PO_2_-dependent regulation of erythrocyte deformability as a key determinant of blood viscosity (Wei et al., 2016).

### Effects of hypoxia on RBC ATP release and purinergic regulation of vascular tone

Besides release of hemoglobin-associated nitric oxide (NO), hypoxia affects vascular tone via release of ATP from RBCs that, in turn, leads to activation of P2Y receptors on endothelial cells, stimulation of NO production and NO-mediated vasodilation (Dietrich et al., 2000; Wang et al., 2005; Ellsworth and Sprague, 2012; for review, see Ellsworth et al., 2009, 2016; Jensen, 2009; Luneva et al., 2015). *In vitro* studies have shown that shear stress, mechanical deformation and hypoxia are major stimuli of RBC ATP release (Bergfeld and Forrester, 1992; Sprague et al., 1996; Forsyth et al., 2012; Mairbäurl et al., 2013). These observations were confirmed using microbore capillaries (Fischer et al., 2003) and microfluidic channels (Price et al., 2004; Forsyth et al., 2011) demonstrating that shear stress *per se* is sufficient to trigger ATP release from RBC (Wan et al., 2011). Importantly, elevated ATP levels have also been found *in vivo* in venous effluent from exercising forearm muscle (Forrester, 1972; Ellsworth et al., 1995) and further augmented by exercise performed in hypoxia (Dietrich et al., 2000; González-Alonso et al., 2002). It has been demonstrated that only when the vessels were perfused with RBCs did venous effluent ATP level increase and the vessels dilate in response to low extraluminal PO_2_ (Dietrich et al., 2000). Recent studies have shown that RBC-mediated ATP release is reduced in aging humans, which may contribute to impaired vasodilation and oxygen delivery to skeletal muscle during hypoxemia with advancing age (Kirby et al., 2012). Attenuated ATP production and release during deoxygenation was also found in banked RBCs, likely contributing to augmented microvascular adhesion of transfused RBCs *in vivo*. These alterations could be substantially corrected by restoring glycolysis-mediated ATP production (Kirby et al., 2014). To the best of our knowledge the comparative analysis of the action of hypoxia on ATP release under baseline conditions and in RBC subjected to shear stress has not been performed yet.

### Search for transporters involved in ATP release triggered by hypoxia

Since mature mammalian RBCs are devoid of intracellular organelles and unable to secrete ATP via endoplasmic reticulum-dependent exocytosis, it might be assumed that ATP release from RBC in hypoxic conditions is mediated by ATP-conducting channels (Praetorius and Leipziger, 2009). Cystic fibrosis transmembrane conductance regulator (CFTR), Pannexin-1 (Panex1), voltage dependent anion channel (VDAC), and other poorly defined VDAC-like maxi anion channels have been implicated in conductive ATP release in RBCs and in other cell types (Sprague et al., 1998; Sridharan et al., 2010, 2012).

Early reports have suggested that CFTR and other members of the superfamily of ATP-binding cassette transport proteins serve as a conductive pathway for ATP release, or regulate an associated ATP channels in several cell types, including RBCs (Reisin et al., 1994; Sugita et al., 1998). Since CFTR activity is regulated by cAMP-dependent PKA, it has been also hypothesized that shear stress- and hypoxia-induced ATP release involves activation of the cAMP signaling pathway (Sprague et al., 2007). Consistent with this hypothesis, ATP release has also been reported in response to other stimuli that elevate cAMP, such as agonists of prostacyclin (Montalbetti et al., 2011; Sridharan et al., 2012), or β-adrenergic receptors (Olearczyk et al., 2001; for review see Ellsworth and Sprague, 2012). However, subsequent studies by several independent groups with the patch clamp, lipid bilayer, and luminometry techniques, have not revealed any detectable CFTR-mediated or CFTR-regulated ATP release in several epithelial and non-epithelial cells (Grygorczyk et al., 1996; Li et al., 1996; Reddy et al., 1996; Grygorczyk and Hanrahan, 1997a,b; Watt et al., 1998; Hazama et al., 1999). In particular, it was also determined that CFTR protein is absent in the RBCs (Hoffman et al., 2004) and the role of cAMP signaling pathway in stimulating RBC ATP release was contradicted by recent studies (Sikora et al., 2014; Keller et al., 2017).

VDAC mediates ATP movement across the outer mitochondrial membrane (Rostovtseva and Colombini, 1997), and similar large conductance anion selective channels have occasionally been found in the plasma membrane of several cells (Báthori et al., 2000). However, these plasma membrane maxi anion channels and VDAC were shown to be unrelated proteins (Sabirov et al., 2006, 2017). VDAC pore selectivity favors the flow of adenine nucleotides (ATP, ADP) and anionic metabolites, over molecules of the same size and charge. The flow of small cations, including Ca^2+^, proceeds at significant rates even for closed channel state (Colombini, 2012). Thus, presence of such large-conductance poorly selective channels in cell plasma membrane would perturb significantly cell homeostasis. Similar concerns may apply to other putative ATP channels (see below).

Connexins and the related pannexins, particularly Panx1 currently appear to be the most extensively investigated family of proteins reported to function as ATP conduits in a broad range of cell types (Romanello et al., 2001; Dando and Roper, 2009; Ma et al., 2009; Ransford et al., 2009; D'hondt et al., 2010; Lazarowski et al., 2011), including RBCs (Sridharan et al., 2010; Qiu et al., 2011; Chu et al., 2016). Nevertheless, several basic Panx1channel properties, including single channel conductance, selectivity and regulatory mechanisms still remain unclear (Chiu et al., 2014). Systematic electrophysiological studies revealed that Panx1 is a relatively low conductance anion channel (unitary conductance of 68 to 75 pS) with negligible permeability to large anions (aspartate, glutamate, gluconate), (Ma et al., 2012; Chiu et al., 2014). In particular, no measurable Panex1 permeability to ATP was detected in taste buds and several heterologous expression systems (HEK-293, CHO, and SK-N-SH cells) (Romanov et al., 2012). Thus, direct patch-clamp experiments to determine its selectivity so far did not provide convincing support for the involvement of these channels in ATP release. It has been suggested however, that permeability characteristics could change depending on how the channel was activated (Chiu et al., 2014).

Piezo 1 is a mechanosensitive non-selective cation channel expressed on RBC membrane. Gain-of-function mutations in Piezo 1 were linked to dehydrated hereditary stomatocytosis (Zarychanski et al., 2012; Albuisson et al., 2013). It was recently shown that Piezo 1 regulates mechanosensitive ATP release in RBCs by controlling the shear-induced Ca^2+^ influx (Cinar et al., 2015). Based on pharmacological data it was proposed that the release may involve CFTR and/or Pannexin 1 channels, but in the light of concerns discussed above, alternative pathways that might be modulated by intracellular Ca^2+^-elevation should be also considered (see below).

Regardless of the molecular nature of ATP-conducting channels, the main conceptual difficulty with such a release mechanism is that ATP permeation requires pore of large dimensions (0.6–1.1 nm, Sabirov and Okada, 2005), resulting in poor selectivity and large conductance (hundreds of pS) for small ions such as K^+^, Na^+^, Cl^−^, and Ca^2+^, as exemplified by the well characterized VDAC channel. VDAC selectivity for ATP, ADP is based on steric constrains and charge distribution allowing to discriminate between large anions but it does not prevent permeation of small cations. Opening of such large, non-selective pores in the plasma membrane will result in significant influx of Na^+^ and Ca^2+^ down their electrochemical gradients into the cytoplasm, posing an overwhelming challenge to normal cell homeostasis and survival (Akopova et al., 2012). Furthermore, in terms of energy expenditure, conductive ATP release would be a costly mechanism of intercellular signaling that requires action of energy-consuming transport processes to re-establish normal cellular Ca^2+^, Na^+^, and K^+^ gradients. To the best of our knowledge, currently there is no example of a channel that on one hand would allow high permeability for anions as large as ATP and, on the other would prevent significant fluxes of small cations. None of the currently known putative plasma membrane ATP channels seems to be compatible with the requirement of preserving cell homeostasis. Therefore, the concept of such release mechanisms should be treated with caution.

### ATP release by hemolysis

**Figure 1:**
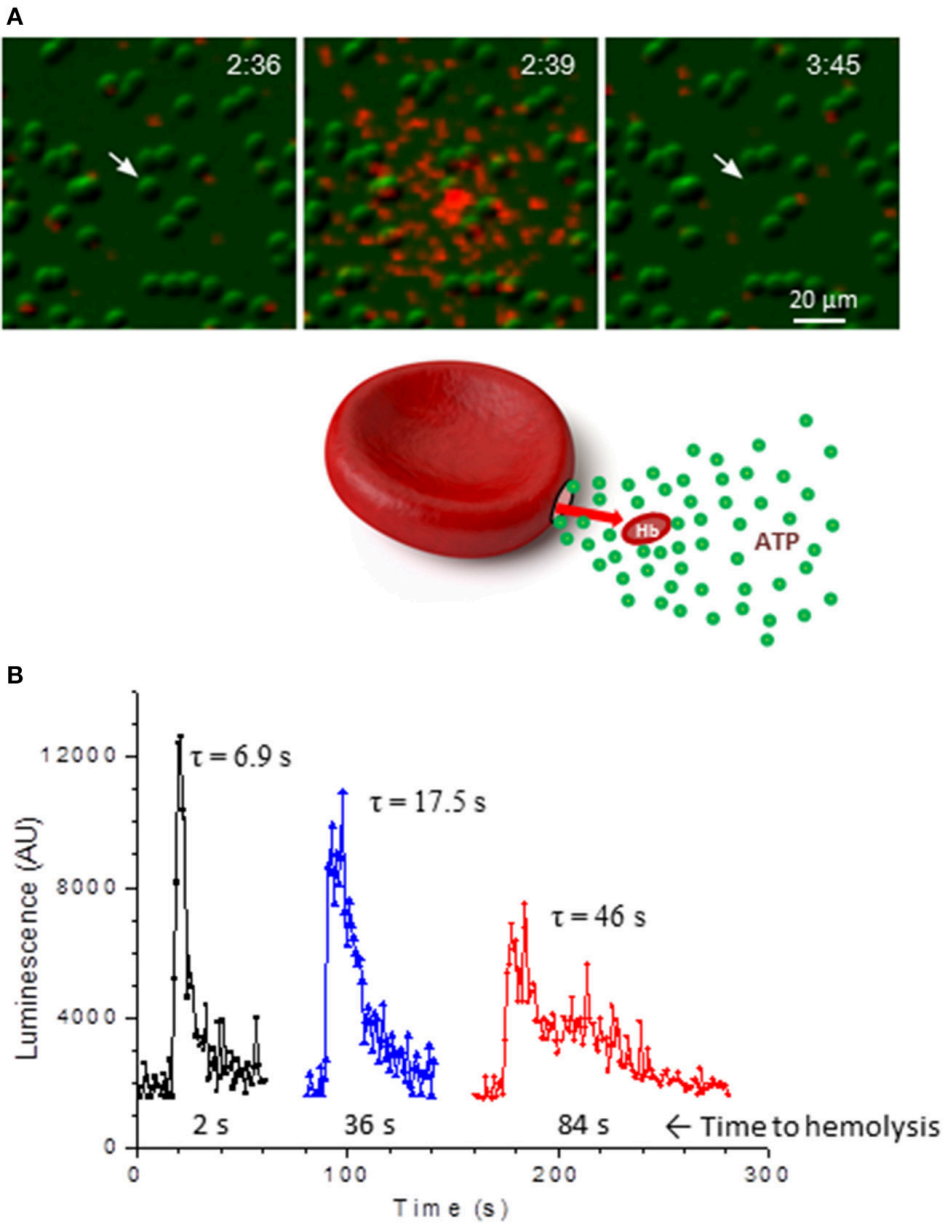
ATP release due to lysis of single RBCs. **(A)**, sequential infrared images of RBCs (green) that are overlaid with extracellular ATP-dependent bioluminescence from luciferin-luciferase reaction (red). Elapsed time is indicated in the right-upper corners (min:s). Twenty percent of hypotonic solution was introduced at time 0 and hypotonic shock-induced ATP release is shown on the center image. Ghosting of the RBC (indicated by arrow) due to Hb leakage occurred with a delay of about 66 s. No ATP release from intact RBCs is evident. **(B)**, three examples of ATP release time-course due to lysis of single RBCs such as shown in A. Duration of ATP release τ (in s) is indicated for each trace and corresponding delay from peak ATP release until cell ghosting is shown below. Adapted from Sikora et al. (2014).

Hemolysis is an important source of extracellular ATP, and intravascular hemolysis occurs *in vivo* as a consequence of hypoxia and mechanical trauma to RBCs (Shaskey and Green, 2000; Mao et al., 2011; Mairbäurl, 2013). Although it has been also considered to be a potential factor contributing to stimulated ATP release in most previous *in vitro* investigations, its actual involvement has often not been assessed systematically. By paired measurements of ATP and free hemoglobin in each and every sample of human RBC supernatants Sikora et al. found that basal and stimulated ATP release not only correlated tightly with extracellular hemoglobin, but matched the levels expected from cell lysis and independently determined cell ATP content (Sikora et al., 2014, 2015). Unexpectedly, this was seen with all stimuli tested (hypotonic shock, shear stress, hypoxia) strongly indicating that, for each stimulus, the only source of extracellular ATP was cell lysis (Sikora et al., 2014; Luneva et al., 2016). Surprisingly, stimulation of cAMP pathway had no effect on RBC ATP release, which remained at the basal level observed with unstimulated cells. The report triggered a significant debate in the field with opposing views presented by Kirby et al. (2015) and Sikora et al. (2015). Absence of cAMP-regulated ATP release was recently confirmed by Keller et al. who also showed using Panex1^−/−^ mice model that Panex1 has no role in exercise performance, challenging assumptions about Panex1 role in ATP-dependent blood perfusion to exercising skeletal muscle (Keller et al., 2017). In the study by Sikora et al. the primary role of hemolysis in hypotonic shock-induced ATP release was confirmed more directly by simultaneous luminescence ATP imaging and infrared imaging of substrate-attached RBCs (Sikora et al., 2014). With luciferin-luciferase (LL) present in the extracellular solution these experiments identified single ATP-releasing cells and revealed that only lysing cells contributed to the release. This was seen as a flash of ATP-dependent LL luminescence around the cell followed, after some delay, with cell ghosting due to Hb leakage, Figure 1A. Individual cells showed variable duration of ATP release as might be expected for different number and/or size of lytic pores in the membrane, Figure 1B. Interestingly, time-course of ATP release correlated with time delay of cell ghosting, i.e., cells showing slower ATP release also displayed longer delay before ghosting, consistent with slower Hb leakage. This is in agreement with 3-to 4-fold difference of their diffusion coefficients in water, supporting the view that release of Hb and ATP proceeds through the common pathway. The study demonstrated that at least in the case of hypotonic shock and cAMP/forskolin stimulation hemolysis is likely the only mechanism of RBC ATP release, and therefore processes that control RBC susceptibility to lysis will also contribute to modulation of ATP release (Sikora et al., 2014). In the light of this finding there is an urgent need to understand mechanisms underlying the hemolysis (Thomas, 2014).

RBC fragility, or propensity to hemolyse under osmotic or mechanical stress, is determined by properties of the cell membrane. It is comprised of a phospholipid bilayer and an underlying two-dimensional cytoskeleton, a network of actin and α- and β-spectrin molecules that are held together by ankyrin. The membrane is stabilized by interactions of ankyrin with band 3, the major RBC membrane integral protein (Mohandas and Gallagher, 2008). The composite properties of the phospholipid bilayer and 2D cytoskeleton are responsible for the biconcave discocyte morphology of healthy RBC and membrane elastic and rheological properties. Disruptions of interactions between cytoskeletal components and/or integral membrane proteins change spectrin network density causing cell morphological changes and membrane fluctuations, affecting RBC deformability and fragility (Diez-Silva et al., 2010).

Under shear stress the RBC cell membrane deforms until the membrane reaches its “yield point.” Beyond this threshold point, additional stress results in irreversible plastic deformation of the membrane, which accelerates with accumulation of microdefects in the membrane, leading to the cell destruction (Orbach et al., 2017). However stressed cells do not rupture immediately, the time-course of this process depends on the duration and extent of the stimuli. Li et al. (2013) showed that after exposure to brief pulse of cavitation-induced shear stress (i.e., during air bubble formation and collapse) RBC lysis is a 2-step process. It involves formation of nanopores followed by colloidal osmotic cell swelling until cell bursts. To reach that point, the nanopores in the membrane must remain for a sufficient time. In erythrocyte membrane the brush-like glycocalyx molecules by steric interactions may contribute to stabilization of nanopores. In their study pores of up to 1.6 μm effective size were formed at rupture site that allowed diffusion of cellular content. Lysis occurred within few seconds to several tenths of seconds. Interestingly, in these experiments cells that ruptured showed irregular shape, indicating that changes in the 2D spectrin network and/or anchor points to plasma membrane are important factors enabling lytic pore formation (Li et al., 2013).

### Role of RBC aging and membrane vesiculation

The circulating RBCs undergo a natural aging process occurring throughout their lifespan of about 120 days. The aged “senescent” cells are characterized by loss of cell surface area, cell morphology alterations, increased cell rigidity and aggregability, reduced level of cell membrane stomatin (band 7 protein), and translocation of phosphatidylserine (PS) to the cell surface. The surface membrane content of sialoglycoproteins and sialic acids which accounts for majority of the negative surface charge of RBC membrane is also reduced in aged RBC contributing to altered RBC membrane mechanical and electrical properties, receptor-mediated cellular interactions, immune responses and survival (Durocher et al., 1975; Huang et al., 2011). The aging of RBCs are also characterized by the formation and accumulation of microdefects in the RBC membrane which as mentioned above, makes them susceptible to stress. Indeed, Orbach et al. found that the cells that were destroyed under low mechanical stress were characterized by low deformability, high level of surface PS, and reduced level of membrane stomatin, all properties consistent with aged senescent cells possessing augmented macrovesicle formation terminated by RBC lysis (Orbach et al., 2017).

The molecular mechanisms underlying the formation of the plasma membrane macrovesicles remain poorly understood. Changes in metabolic status and decrease of cellular ATP levels during prolonged deoxygenation induce RBC shape changes and increase membrane fluctuations. Membrane fluctuations are directly linked to binding of membrane bilayer to spectrin network which is actively controlled by ATP (Diez-Silva et al., 2010; Park et al., 2010). Low ATP-induced morphological changes are reversible upon restoration of normal cellular ATP levels. Thus, metabolic status of RBC might be important factor affecting RBC susceptibility to membrane vesiculation and RBC lysis. It was also well documented that RBC vesiculation is sharply potentiated by elevation of intracellular Ca^2+^ concentration ([Ca^2+^]_i_) via activation of scramblase and inhibition of flipase. These [Ca^2+^]_i_-dependent events results in a collapse of membrane phospholipid asymmetry and cytoskeleton detachment (for review see (Greenwalt, 2006; Alaarg et al., 2013). In early studies, Tiffert and co-workers observed that brief deoxygenation results in elevation of [Ca^2+^]_i_ up to 70% that was probably caused by Ca^2+^-ATPase inhibition (Tiffert et al., 1993).

Intravascular vesiculation process and associated hemolysis of senescent cells in healthy subjects was reassessed in recent study by Ciana et al. (2017). They showed that contrary to some earlier *in vitro* investigations vesiculation process of senescent RBCs removes membrane in a balanced way as a lipid bilayer vesicles containing membrane cytoskeleton. Moreover, the study suggests that *in vivo* vesiculation almost entirely occurs by active processing in the spleen producing progressively smaller but otherwise viable discoid shape cells. This agrees with the view that vesiculation is a self-protective mechanism to remove damaged membrane patches containing removal proteins, thereby postponing untimely elimination of healthy RBCs (Willekens et al., 2008). The study implies that in healthy subjects under normal conditions contribution of intravascular hemolysis to RBC clearance may be negligible. Therefore, intravascular hemolysis may occur only under particular conditions in the localized regions of the vasculature where elevated shear and hypoxia may arise, such as in the microvessels of skeletal muscle during intense exercise. It should be noted, however, that due to high ATP content of RBCs (1–5 mM) even negligibly small intravascular hemolysis may readily produce local ATP concentrations reaching ~1 μM, sufficient for purinergic control of blood flow. For example, lysis of a single erythrocyte will result in ATP concentration of 1 μM within 2–10 mm long segment of a capillary with a diameter comparable to RBC size (7 μm). Thus, a miniscule fraction of the circulating pre-senescent cells, e.g., those prior to their processing in the splenic system could constitute a sufficient pool of RBCs available for intravascular hemolytic ATP release and blood flow control. Contribution of the oldest RBCs showing so called terminal density reversal and the role of the nonselective cationic channels in the sustained elevation of Ca^2+^ and triggering of hemolysis should be also considered (Lew and Tiffert, 2013; Thomas, 2014).

### Search for upstream PO_2_ sensors and downstream intermediates of PO_2_-dependent signaling

Hemoglobin is the only known O_2_-binding protein in erythrocytes. Keeping this in mind, the reversible association of oxygenated and/or deoxygenated hemoglobin (oxyHb and deoxyHb, respectively) with downstream intermediates of intracellular signaling might be considered as a mechanism of triggering PO_2_-dependent erythrocyte responses. Indeed, in cell-free experiments it was shown that hemoglobin binds to the cytoplasmic domain of band 3 (cdb3) (Cassoly, 1983; Low et al., 1984) also known as anion exchanger (AE1, SLC4A1), i.e., the major integral protein of erythrocytes membrane, playing a key role in anion transport and the organization of membrane cytoskeleton (Reithmeier et al., 2016). Importantly, both in human and mice the affinity of cdb3 for deoxyHb is much higher than for oxyHb (Walder et al., 1984; Sega et al., 2012, 2015).

**Figure 2:**
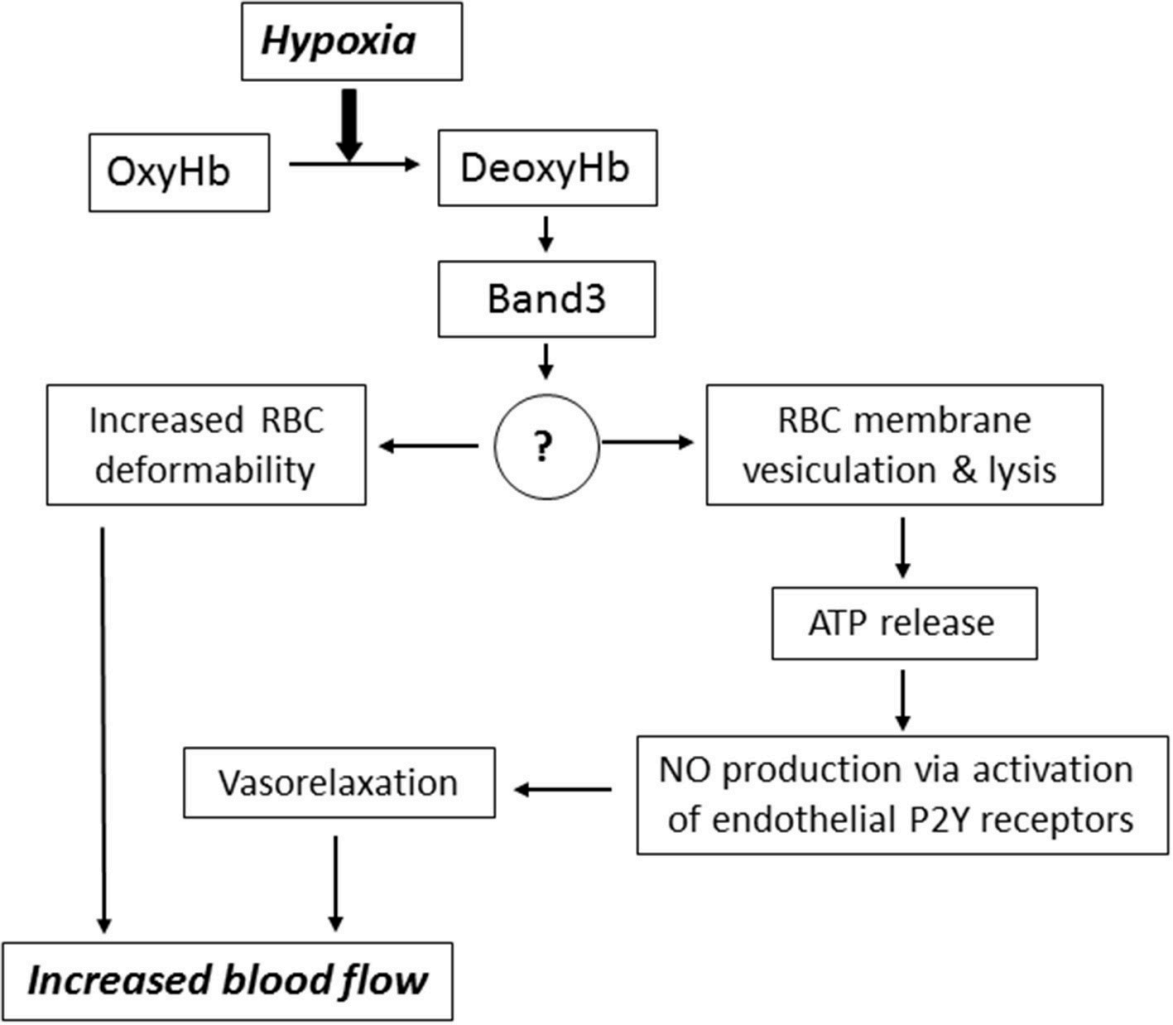
Mechanisms underlying the implication of RBC in blood flow regulation. Hypoxia leads to accumulation of deoxyhemoglobin (DeoxyHb) and its interaction with cytoplasmic domain of anion exchanger (Band 3 protein). This interaction triggers signaling via changes in the content of unknown membrane-bound proteins (?) resulting in increased RBC deformability, membrane vesiculation and ATP release via hemolysis. For more details, see text.

Downstream intermediates of PO_2_-dependent erythrocyte responses remain poorly understood (Figure 2). Stefanovic and co-workers has demonstrated that oxygentation strengthens band 3-ankyrin interactions, thereby stabilizing the erythrocyte membrane during turbulent flow from the lungs to the capillary beds (Stefanovic et al., 2013). Deoxygenation displaces ankyrin from band 3 releasing the spectrin/actin cytoskeleton from the membrane. This weakening of membrane-cytoskeleton interactions could increase RBC deformability enabling deoxygenated RBCs move more efficiently through narrow capillaries. Prolonged deoxygenation, on the other hand, could increase membrane propensity to rupture due to diminished mechanical support by the cytoskeleton. Indeed, theoretical considerations of membrane stability and pore formation in a lipid bilayer showed importance of cytoskeletal network in stabilizing the membrane against pore growth by reducing the surface tension (Sung and Park, 1997).

It is important to note that when oxygenated RBCs enter a region of low PO_2_ the full-scale saturation of hemoglobin deoxygenation occurs within 25 ms (Ellsworth et al., 2016). Therefore, we constructed a special chamber allowing isolation of RBC ghosts in control and deoxygenated conditions and found a ~2-fold elevation of ~60 kDa membrane-bound protein content under deoxygenated conditions (Luneva et al., 2016). Currently, we employ proteomics technology for identification of full set of proteins whose interaction with RBC membrane is affected by hypoxia. This approach should lead to identification of downstream intermediates involved in hypoxia-induced ATP release mediated by diminished RBC membrane integrity.

## Future research directions

Several issues remain unsettled and require further investigations. Among them the question remains if besides hemolysis as the primary release mechanisms, are there conditions or stimuli that would induce regulated non-lytic ATP release from RBC and what pathway it could involve? What might be the contribution of young immature erythrocytes (reticulocytes) to such release? With their residual intracellular structures could they release ATP via exocytosis? While modest in relative terms (0.5–2.5%) these cells constitute a significant cellular pool (~4.5 × 10^4^ cells/μl) with plenty of ATP for local purinergic signaling. If conductive release pathway would be involved what could be the mechanism allowing selective permeation of ATP but preventing massive influx of Ca^2+^ and Na^+^? Finally, much remains to be learned about the downstream intermediates involved in hypoxia-induced membrane fragility, membrane microdefects, lytic pore formation and associated ATP release.

## Author contributions

All authors listed have made a substantial, direct and intellectual contribution to the work, and approved it for publication.

### Conflict of interest statement

The authors declare that the research was conducted in the absence of any commercial or financial relationships that could be construed as a potential conflict of interest.
